# The influence of “bad news” and “neutral/good news” on patients' perception of physician empathy during oncology consultations

**DOI:** 10.1002/cam4.6903

**Published:** 2024-01-01

**Authors:** Mattias Tranberg, Henrik Ekedahl, Carl Johan Fürst, Jacob Engellau

**Affiliations:** ^1^ Division of Palliative Care, Department of Clinical Sciences Lund Lund University Lund Sweden; ^2^ The Institute for Palliative Care at Lund University and Region Skåne Lund Sweden; ^3^ Department of Oncology Skåne University Hospital Lund Sweden

**Keywords:** bad news, CARE measure, empathy, oncology, patients, physicians

## Abstract

**Objectives:**

Being met with empathy increases information sharing, treatment coherence, and helps patients to recover faster. However, we do not know how the content of the conversation about disease progression, new treatments, or other issues concerning serious illness affects patients' perceptions of the physician's empathy, and thus, the quality of the conversation. This study aimed to test the hypothesis that patients will rate their physician lower following a “bad news” consultation using the consultation and relational empathy (CARE) measure.

**Methods:**

A total of 186 outpatients from the Department of Oncology were recruited for this study. After meeting with a patient, the physician filled out a form, placing the patient in either the “bad news” group, or the “neutral/good news” group along with information about the patient and the consultation. The patient was given the CARE measure after the visit.

**Results:**

The patients who had received bad news rated their physicians a significantly lower score on the CARE measure, even though the effect size was small, than those who had neutral/good news. On average, bad news consultations were 11 min longer.

**Conclusions:**

Physicians need to be aware of the patients' need to be known and understood, in addition to having skills to attend to emotional cues and concerns, since the current study's finding could be a sign either of the content being projected onto the physician or that the physician is focused on the message rather than on the patient.

## Abstract 2

This article describes a comparison in perceived physician empathy between groups of patients with cancer who had either bad news or neutral/good news consultations. The results indicate a difference, which highlights the importance for physicians to make sure patients feel heard and understood.

## 1 BACKGROUND

Breaking bad news in oncology is associated with significant distress for both patients and physicians,^1^ as the topic of the conversation is a life‐threating disease. Hence, there is a risk that the patient will associate the message with the messenger, perceiving the physician as less empathic and having less benign intentions, when delivering information with negative and threatening content.^2^ This is contrary to the goal of any physician and the needs of patients, who wish to be met with empathy and understanding.^3^

Empathy and compassion in healthcare have increasingly gained attention from researchers, clinicians, patients, and families as being important to quality of care. Empathy has been discussed and attempted to be defined over a long period of time; there seems to be a shared view that, empathy means that the empathizer understands, feels, and shares the other person's feelings while being mindful of the separation between the self and the other.^4^ Compassion requires empathy and goes one or two steps further in including a desire, and in some definitions, also including action, to prevent or alleviate the other's suffering. Verifying the patient's understanding is part of achieving empathic accuracy and allows the patient to feel known and understood.^5^

Empathy is thought to impact patient outcomes such as satisfaction, compliance, trust, health status, psychological state, enablement, and self‐management through three different processes.^6^ The first is building a relationship through small talk and friendly gestures to establish trust, and the second is an affective process where physicians listen to what is important to the patient and show compassion for reactions and concerns. The third process is cognitive, which rests on understanding patient needs and is action‐oriented, such as proposing adapted therapeutics and helping patients take control.^7^ Communication is important in all forms of healthcare, and a study of 266 complaints revealed that patients in oncology had more concerns about relationships and communication than over technical aspects of care.^8^

We know that being met with empathy increases information sharing and treatment coherence, and even helps patients recover faster.^9^ However, we do not know how the content of the conversation about disease progression, new treatments, or other issues concerning serious illness affects patients' perceptions of the physician's empathy, and thus, the quality of the conversation.

The primary aim of this study was to test our hypothesis, that patients would experience less empathy from the physician after a consultation with breaking bad news compared to a more neutral consultation. The secondary aim was to test, whether the length of consultation and familiarity with the physician affected the perceptions of empathy.

## 2 METHODS

The inclusion criterion for physicians was that, they were treating patients with cancer in an outpatient hospital setting. The inclusion criteria for patients were outpatients over 18 years of age with any kind of cancer and awareness, that they had cancer. Patients who had their first consultation at the oncology clinic were excluded, because it was difficult to determine what they knew about their disease in advance and, thus, it was difficult to know what group (Table 1) to place them in. Seventeen physicians and 233 outpatients were recruited for the study. Data were collected between October 2019 and January 2022.

**TABLE 1:** Criteria for neutral/good news and bad news consultations.

Neutral/good news consultation	Bad news consultation
Disease is stable or no evidence of disease during follow‐up without treatment	Relapse during maintenance treatment or follow‐up
Stable disease or regression during oncological treatment	Relapse during adjuvant treatment
Planned visit during treatment without imaging prior to visit and without reported grade 3 or 4 adverse events	Progression during treatment of manifest disease
Follow‐up visit 3 months after radiotherapy	New discovery or symptom that requires investigation of a suspected relapse/progression

This study was conducted using self‐report questionnaires. Physicians working in the Department of Oncology, Skåne University Hospital, Sweden were invited to participate in the study. An assistant nurse approached the patient in the waiting room ahead of the consultation. The patients were given a detailed oral and written study description, an informed consent form for them to sign if they wanted to participate, the questionnaire, and a stamped envelope. If they had any questions, they were encouraged to contact the research team. If they consented to participate, they were invited to fill out the questionnaire, sign the letter of consent, and post the envelope after returning home. After the consultation, the physician filled out a form, stating the main content of the consultation (Table 1). The different topics of consultations were pre‐specified as either “bad news consultations,” or “neutral/good news consultations.” The form also included medical and clinical data—age, sex, marital status, whether the patient was accompanied by a close relative, type of cancer, treatment intent (curative/palliative), length of visit, and how well the physician knew the patient.

Patient perception of physician empathy was measured using the consultation and relational empathy (CARE) measure. The CARE measure was developed for use in primary care.^10^ The scale evaluates the patients' perception of their referent physicians' empathy at the last consultation using 10 items. The scale has a five‐point Likert response scale: “Poor, Fair, Good, Very Good and Excellent” to the question “How was your doctor at….” It also proposes a “not applicable (N/A)” answer. Up to 2 N/A responses or missing values were allowed and replaced with the average item score. The scores ranged from 10 (low perceived empathy) to 50 (high perceived empathy). Cronbach's alpha for the original version of the scale was *α* = 0.92.^10^

The CARE measure can be divided into two subscales, the “listening/compassion” subscale consisting of item 1–6 and the “active/positive empathy” subscale consisting of item 7–10.

The listening/compassion part of perceived empathy deals with patients feeling comfortable with their physician, the physician showing attention, and compassion toward patients' difficulties and concerns. In response, patients feel that they are listened to, treated as individuals, and acknowledged in their difficulties.

In active and positive empathy, which is more of a cognitive process, the physician shows empathy by supplying information and options, and is more oriented toward an action plan.

The CARE measure has been successfully used in the oncology setting before^7^, ^11^, ^12^, ^13^, ^14^ with mean scores ranging 37.14–43.2, but not in Swedish oncology. However, it has been translated into Swedish and validated in primary care with a mean score of 41.5 (SD 8.9), and a Cronbach's alpha of 0.975, and neither age nor gender had a significant influence on the results.^15^

We tested the reliability of the CARE measure by calculating Cronbach's alpha. As we suspected, the distribution of the score of the CARE measure was negatively skewed, because of the well‐known ceiling effect of the CARE measure^15^, ^16^, ^17^ and the distribution of length of consultation was positively skewed; therefore, we used nonparametric tests. Mann–Whitney's *U* ‐test and Kruskal–Wallis *H*‐test were used to analyze the differences between the groups regarding the CARE measure score, level of physician familiarity with the patient, and length of consultations. We used the standardized effect size r (*Z*/√(*n*1 + *n*2)) to determine the effect strength. Pearson's chi‐squared test was used to determine the goodness of fit between the groups in relation to the level of physician familiarity with the patient. Spearman's correlation was used to analyze the correlation between the length of consultation and the CARE measure score. All tests were two‐tailed, included outliers, and used an alpha level of 0.05.

Statistical analyses were performed using IBM SPSS Statistics version 28 (IBM Corp., Armonk, NY, USA).

## 3 RESULTS

Of the 233 patients who were invited to participate, 191 signed consent forms and filled‐in questionnaires were received, yielding a response rate of 81.9% (83.7% for the “bad news” group vs. 81.6% for the “neutral/good news” group). Five questionnaires had more than two missing items and were excluded from the study, resulting in 186 properly rated consultations. There were 155 (83%) consultations with “good/neutral” information and 31 (17%) “bad news” consultations (Table 2).

**TABLE 2:** Patient demographics.

	Total (*n* = 186)	Neutral/good news group (*n* = 155)	Bad news group (*n* = 31)
Gender			
Female	77 (41%)	57 (37%)	20 (64%)
Male	109 (59%)	98 (63%)	11 (36%)
Age (years)			
Mean	67	67	66
Range	28–91	28–86	42–91
Marital status			
In a relationship	142 (76%)	118 (76%)	24 (77%)
Single/widowed	44 (24%)	37 (24%)	7 (23%)
Came to consultation			
Alone	121 (65%)	100 (65%)	21 (68%)
With company	65 (35%)	55 (36%)	10 (32%)
Treatment intention			
Curative	116 (63%)	104 (67%)	13 (42%)
Palliative	69 (37%)	51 (33%)	18 (58%)
Diagnosis			
Prostate cancer	62 (33%)	61 (39%)	1 (3%)
Malignant melanoma	44 (24%)	32 (21%)	12 (39%)
Breast cancer	40 (22%)	29 (19%)	11 (35%)
Other malignant disease	40 (22%)	33 (21%)	7 (23%)

The median score of the CARE measure was 48, with a range of 10–50 and the Cronbach's alpha was 0.958. Patients rated their physicians with a significantly lower score on the CARE measure if they had received bad news (median 44) than if they had received neutral/good news (median 48, *p* = 0.044, Table 3). The effect size of this analysis was small (0.14). Regarding the subscales of the CARE measure, patients who had received bad news rated their physician significantly lower than those who received neutral/good news did for “Listening/compassion” (median 27 vs. 29, *p* = 0.026) with an effect size of 0.16, but not regarding “Active/positive empathy” subscale (median 17 vs. 20, *p* = 0.063).

**TABLE 3:** The median scores on the CARE measure and time of consultation.

	Total	Good/neutral news	Bad news	Difference between groups
The CARE measure	Median score (range, interquartile range)	Median score (range, interquartile range)	Median score (range, interquartile range)	Effect size (*p*‐value)
Full scale^a^	48 (10–50, 41–50)	48 (29–50, 41–50)	44 (10–50, 37–50)	0.15 (0.044*)
Active/positive empathy subscale^a^	19 (4–20, 26–20)	20 (11–20, 16–20)	17 (4–20, 14–20)	0.14 (0.063*)
Listening/compassion subscale^a^	29 (6–30, 25–30)	29 (18–30, 25–30)	27 (6–30, 23–30)	0.16 (0.026*)
Length of consultation (min)	Time (range, interquartile range)	Time (range, interquartile range)	Time (range, interquartile range)	Effect size (*p*‐value)
Median^a^	30 (7–60, 25–45)	30 (7–60, 20–40)	45 (20–60, 34–45)	0.33 (<0.001**)

^a^ Mann–Whitney *U*‐test.

* *p* < 0.05.

** *p* < 0.001.

The median length of consultations was 30 min, with a range of 7–60. Consultations including “bad news” were significantly longer than “neutral/good” consultations (median of 45 min compared to 30 min, *p* < 0.001, and a medium effect size of 0.33). There was a significant positive correlation between the length of the consultation and the CARE measure score (*r* = 0.14513, *p* = 0.04872), indicating that the longer the consultation, the higher the score.

There was no significant difference between the groups regarding physicians' familiarity with the patient (*p* = 0.900), and the level of familiarity did not have a significant effect on the score of the CARE measure (Table 4). Four instances of missing data were excluded from analysis.

**TABLE 4:** Physician familiarity with the patient.

How well did the physician know the patient? *N* (%)	Not at all	A little	Well	Missing data	Effect size (*p*‐value)
Total	113 (61%)	53 (29%)	16 (9%)	4 (2%)	(0.900)^a^
Good/neutral news	94 (61%)	44 (28%)	14 (9%)	3 (2%)	
Bad news	19 (61%)	9 (29%)	2 (6%)	1 (3%)	
The CARE measure^b^					
Median score (range)	48 (10–50)	46 (31–50)	50 (33–50)		−0.0024 (0.4564)

^a^ Pearson chi‐squared test “A little” grouped with “Well” compared with “Not at all.”

^b^ Kruskal–Wallis *H*‐test.

## 4 DISCUSSION

In this study, while the effect size was small, we found that patients perceived physicians who delivered bad news as less empathic than those who delivered neutral or good news. Patients did not feel that the physicians listened to and expressed compassion to the same extent when breaking bad news. However, the participating oncology physicians were generally perceived as highly empathic compared to other studies in oncology settings^6^, ^11^, ^12^, ^13^, ^14^ and primary care in Sweden.^15^ The length of the consultation had a weak but positive impact on perceived physician empathy, which did not compensate for the impact of the content. How well the physician and patient knew each other, that is, the continuity of care, did not have a significant impact.

It could be proposed that patients' perception of their physician as less empathic after receiving bad news is an example of “shooting the messenger,”^2^ or that the patient idealizes the physician after having received good news after a period of worry in anticipation of the visit. One explanation for the lower rating following a bad news consultation could be that the physician focuses on the message^18^ and, as a result, pays less attention to the patient, which is counterproductive. For the patient, it is as important, if not more important, to be known as it is to know what is ahead. It could also be a sign of the physician feeling distress over disclosing bad news, wanting to move ahead to the solution, which would be measured by the items in the subscale “Active/positive” empathy. These suggestions align with findings from a study on communication with patients with breast cancer, where the physician emphasized the severity of the illness more than the physician–patient relationship when the expectations were bad, compared to when expectations were good.^19^ Although we did not measure physician stress in this study, Neumann et al.^13^ cited studies that showed that stress has a negative effect on residents' empathy and reduces the signal rate of mirror neurons. In a recent study of oncology patients' perspective on empathy, patients valued “Listening” the most, followed by “Attention to what matters most to me” and “Demeanor.”^20^ The “Listening/compassion” score of the CARE measure consists of items that quantify paying attention to and caring for the patient, such as “How was the physician at being interested in you?” and “How was the physician at showing care and compassion?” which corresponds to the previously mentioned study. Another recent study showed that, among potentially harmful behaviors, making vague promises, and not listening to the patient were considered the worst.^21^ In that study, 65% of the patients thought that too little information was harmful, and 60% thought too much information was harmful, which indicates the difficulty of getting it right. This highlights the need for careful questioning and listening to patient preferences.

The length of consultations may partly explain why physician empathy was perceived as higher than in previously published studies in both primary care in Sweden and oncology settings.^7^, ^11^, ^12^, ^13^, ^14^, ^15^ The cited studies using the CARE measure did not report the length of consultations, but in other studies in those settings, the consultations averaged 22.5 and 22.9 min, respectively.^22^, ^23^ It was surprising that the length of the consultation did not have a higher impact on perceived empathy, since the physician showed the intent of wanting to care for patients receiving bad news, from the perspective of allocating more time with the patient. Further studies are warranted to explore the interaction between time and communication skills, and how it affects perceived empathy in bad news consultations. Another aspect of physician–patient interaction is how the meeting is characterized in terms of the distribution of time spoken. This was not addressed in the current study, but it could be envisaged that a physician under emotional stress resorts to controlling the situation by providing more information^24^ and thus, not paying attention to emotional cues from the patient. There is some evidence that communication skills training improves empathy and reduces the risk of healthcare practitioners providing facts only.^25^

In a previous study, where the physicians knew the patients well there was no perceived difference in physician empathy between groups of bad news consultations or good/neutral news consultations.^11^ Discontinuity of care is linked to negative patient outcomes,^26^ and oncologists have been shown to care more about patients' quality of life when they know them well.^27^ However, in this study, the patient's previous relationship with the physician did not have a significant impact on perceived empathy, regardless of what group the consultation belonged to, which could be due to the small number of consultations where there was a previous relationship. One reason for the low proportion of physicians, who knew the patient well could be that an existing relationship would make both the patient and physician more comfortable having the consultation by phone due to the pandemic, rather than meeting in person. Another explanation could be that although relational continuity, which is the most valued,^28^ was lacking for many patients, the physicians compensated for it through informational continuity and management continuity. That is, information about the patient's medical history and ongoing interventions were available to the physician going into the consultation, thereby accommodating the patient's need to be known and providing adequate services.

### 4.1 Study limitations

One limitation of the study was that, we used an instrument that is intended to measure empathy from the patient's perspective, but was developed through feedback from primary care clinicians without input from patients. A wider take on empathy, based on patients' definitions, might have provided more information about the encounters and how the level of acquaintance between patients and physicians failed to have a significant effect on perceived empathy.

A second limitation of this study was that, we used a subjective assessment of physician familiarity with the patient rather than the number of previous encounters. For one physician, meeting a patient three times may signify knowing them “a little,” while another physician might assess that as knowing them “well.”

The ceiling effect also limits the extent of the findings, the generally very high ratings in this study make it difficult to differentiate between content. The Chinese CARE measure has uniquely not shown a ceiling effect,^29^ which might be indicative of a more developed review culture.

Another limitation of the study is that it was conducted during the COVID‐19 pandemic, which had a negative impact on the number of patients recruited. Many patients who had a stable disease and had met the physician before had their consultation by phone and were excluded from participation. While limiting the recruitment of participants, this circumstance adds an insight to the impact of COVID‐19.

A strength of this study is the high response rate among both groups,^30^, ^31^ which indicates that the selection is probably a fair representation of the study population and shows the importance of empathy for the patient.

### 4.2 Clinical implications

Physicians need time to plan and practice ways to break bad news, encouragement to focus on the patient, and listen to what matters to them. Communication skills training might be a way to help physicians become more aware of and able to reflect on their own behaviors and emotions during consultations as well as on the patient's.

## 5 CONCLUSIONS

Physicians need to be aware of the patients' need to be known and understood, in addition to having skills to attend to emotional cues and concerns, since the current study's finding could be a sign either of the content being projected onto the physician or that the physician is focused on the message rather than on the patient.

## AUTHOR CONTRIBUTIONS

**Mattias Tranberg:** Conceptualization (lead); investigation (equal); writing – original draft (lead). **Henrik Ekedahl:** Formal analysis (equal); methodology (equal); resources (supporting); writing – review and editing (supporting). **Carl Johan Fürst:** Conceptualization (equal); methodology (equal); writing – review and editing (equal). **Jacob Engellau:** Resources (equal); supervision (lead); writing – review and editing (equal).

## CONFLICT OF INTEREST STATEMENT

The authors declare no conflicts of interest.

## ETHICS STATEMENT

The study was approved by the Swedish Ethical Review Authority (approval number: 2018/826).

## Data Availability

The data that support the findings of this study are available on request from the corresponding author. The data are not publicly available due to privacy or ethical restrictions.
